# Repeatability and reproducibility of applanation resonance tonometry: a cross-sectional study

**DOI:** 10.1186/s12886-015-0028-9

**Published:** 2015-04-10

**Authors:** Laura Ottobelli, Paolo Fogagnolo, Paolo Frezzotti, Stefano De Cillà, Elena Vallenzasca, Maurizio Digiuni, Ruggiero Paderni, Ilaria Motolese, Simone Alex Bagaglia, Eduardo Motolese, Luca Rossetti

**Affiliations:** Eye Clinic, Dipartimento di Scienze della Salute, San Paolo Hospital, University of Milan, Milan, Italy; Dipartimento di scienze oftalmologiche e neurochirurgiche, Università degli Studi di Siena, Siena, Italy; Eye Clinic, Azienda Ospedaliero-Universitaria Maggiore della Carità, Novara, Italy

**Keywords:** Applanation resonance tonometry, Glaucoma, Goldmann applanation tonometry, Repeatability, Reproducibility

## Abstract

**Background:**

To assess repeatability (intra-observer variability) and reproducibility (inter-operator variability) of intraocular pressure (IOP) measurements with servo-controlled Bioresonator Applanation Resonance Tonometry (ART) and to evaluate possible influential factors.

**Methods:**

The study included 178 patients (115 glaucoma and 63 controls; one eye per subject). IOP was measured once with a Goldmann applanation tonometer (GAT) and twice by ART (ART1, ART2), in randomized sequence, by a single operator to assess intra-operator variability. Each ART measurement consisted on 3 readings. To assess inter-operator variability 2 evaluators performed 2 measurements each (in random order) on the same patient. Repeatability and reproducibility were assessed by the coefficient of variation (CoV) and intraclass correlation coefficient (ICC).

**Results:**

In the entire cohort, ART1 was 0.4 ± 2.2 mmHg (−7.0 to 5.7 mmHg) higher than ART2 (p = 0.03) regardless of test order. Intra-operator CoV was 7.0% ± 6.3%, and ICC was 0.80-0.92. Inter-operator CoV ranged between 5.7% ± 6.1% and 8.2% ± 7.2%, and ICC between 0.86 and 0.97. ART1 and 2 were respectively 1.7 ± 3.1 and 1.3 ± 3.1 mmHg higher than GAT (p < 0.01). Test-retest difference with ART fell within ±1 mmHg in 41% of cases, within ±2 mmHg in 70%, within ±3 mmHg in 85%. 15% had a test-retest difference higher than ± 3 mmHg; Bland-Altman 95% intervals of confidence were −3.9 and +4.6 mmHg. Results were unaffected by age, diagnosis, central corneal thickness, keratometry, operator, randomization sequence.

**Conclusions:**

In most cases ART repeatability and reproducibility were high, with no differences due to patients’ characteristics. ART measurements overestimated GAT by a mean of 1.3-1.7 mmHg.

## Background

High intraocular pressure (IOP) is the most important and the only modifiable risk factor for glaucoma development and progression [1]. Therefore, accurate IOP estimation is crucial for proper management of glaucoma patients [2,3]. Goldmann applanation tonometry (GAT) is the gold standard for IOP measurement but is limited by ocular properties, including central corneal thickness, corneal shape (radius and astigmatism), axial length and variations in corneal biomechanics [4-6]. The ideal tonometer is expected to be accurate, repeatable, reproducible, and minimally influenced by corneal properties and variability due to examiners. New slit-lamp mounted tonometers that may be less influenced by sources of error have been recently developed, and applanation resonance tonometer (ART) is one of these. ART is a multi-point method based on resonance technique: the contact area between sensor and cornea is measured with a piezoelectric element oscillating in its resonance frequency producing a frequency shift proportional to the contact area. A continuous and simultaneous sampling of both contact area and contact force through a force transducer enables the calculation of IOP according to Imbert-Fick’s law [2,7-9]. ART has been shown to be less affected by corneal properties than GAT thanks to the convex tip and the continuous simultaneous multipoint sampling of both parameters included in the applanation principle: force and area [2,10,11].

The ART system has been evaluated in *in vitro* porcine-eye set-ups using a bench-based horizontal model [2] and a biomicroscope-based vertical model [12]. In the bench-based model, ART showed high precision as compared with the reference IOP (IOP measured in the vitreous chamber)_:_ ±0.94 mmHg (SD) [2]. Slit-lamp measurements (both *in vitro* and *in vivo*) obtained lower precision due to the possible off-centered placement of the sensor during the procedure [12,13]; technical implementations have been introduced to reduce the off-centre dependency [14,15]. In a clinical study on humans, ART precision was slightly better using the biomicroscope set-up as compared with the handled one (SD of 2.07 mmHg and 2.50 mmHg, respectively) [3]. In recent years, further improvement was obtained by adopting an automatic servo-controlled system, which showed overall similar results to the manual one, except for a lower performance at high IOP [14].

To our knowledge, there are only two prospective single-centre clinical studies in literature comparing ART to GAT: the first study did not show significant difference in accuracy between the two methods when IOP <23 mmHg [14] whereas the second study assessed ART repeatability was excellent but significantly lower than that of GAT and ART significantly overestimated GAT measurements, especially at higher IOP range [16].

The aim of the present study was to assess repeatability (intra-observer variability) and reproducibility (inter-observer variability) of IOP measurements with the new servo-controlled Bioresonator ART® (Bioresonator Good Eye AB, Umeå, Sweden) in patients with primary open-angle glaucoma (POAG) and controls, and to assess whether eye parameters and the sequence of the measurements may influence ART readings.

## Methods

This was an observational, cross-sectional study involving two Italian sites: Eye Clinics of San Paolo Hospital of Milan and University of Siena. The study protocol was in adherence to the tenets of the Declaration of Helsinki, it was approved by the Ethics Committees of both centres, and all participants provided written informed consent before enrolment. The study included one eye from 178 consecutive subjects from January to March 2012, including 115 patients with POAG, and 63 controls, whose characteristics are given in Table 1. One eye per patient was randomly selected if both eyes met the inclusion criteria. Normal eyes were recruited from hospital staff, relatives and normal subjects undergoing routine refraction examination; POAG patients were recruited from the Glaucoma Units.

**Table 1:** **Demographic and ophthalmic characteristics of study participants**

	**Overall**	**Normal subjects**	**Glaucoma subjects**	***p***
No of subjects	178	63	115	
Age,mean ± SD (range),years	69.0 ± 9.5 (23 to 92)	68.9 ± 9.6 (34 to 84)	69.1 ± 9.4 (23 to 92)	0.91
Sex (F/M)	94/84	36/27	58/57	
Keratometry, mean ± SD (range), dioptres	44.14 ± 1.78 (37.83 to 48.67)	43.90 ± 2.07 (37.83 to 48.67)	44.27 ± 1.61 (41.39 to 47.96)	0.26
Axial legth, mean ± SD (range), mm	23.48 ± 1.41 (21.17 to 29.67)	23.59 ± 1.68 (21.65 to 29.41)	23.43 ± 1.25 (21.17 to 29.67)	0.51
Central corneal thickness, mean ± SD (range), μm	543 ± 31 (444 to 634)	547 ± 27 (489 to 623)	540 ± 33 (444 to 634)	0.18

The inclusion criteria were: best-corrected visual acuity (BCVA) of 20/30 or better; 18 years old or older; and transparent ocular media (lens opacity < 1 according to Lens Opacities Classification System III system). Subjects with any of the following exclusion criteria were rejected: corneal pathology or contact lens wearers; history of intraocular surgery (i.e. cataract) in the past six months; history of corneal refractive surgery; secondary causes of glaucoma; keratoconus; nystagmus; and neurological disorders. At a screening visit, all patients underwent a full ophthalmological examination to confirm diagnosis, which included: medical history, biomicroscopy, gonioscopy, GAT (Haag-Streit International, Koniz, Switzerland) and indirect fundus ophthalmoscopy. Visual field examination was performed using Humphrey Field Analyser (HFA) II 750 (Carl Zeiss Meditec, Dublin, California) 30–2 test with Fast Swedish Interactive Threshold Algorithm (SITA) strategy. POAG eyes were defined as having: IOP >21 mmHg prior to medication, glaucomatous optic neuropathy (increased optic nerve head excavation due to undermining or notching of the neural rim, disc hemorrhage, focal or generalized atrophy of the nerve fiber layer) and repeatable abnormal visual-field results. During the study, all POAG patients were receiving medical treatment to reduce IOP. Control subjects were defined as having an IOP ≤21 mmHg with no ocular pathologies or signs of glaucoma.

### Ethics statement

Ethics Committees of San Paolo Hospital of Milan and Ospedale Policlinico Santa Maria delle Scotte of Siena approved the study.

### Study procedures

Each subject enrolled in the study underwent the following, in respective order: BCVA evaluation, keratometry and biometry by optical biometry system (IOL Master; Carl Zeiss AG, Feldbach, Switzerland), pachimetry by rotating Scheimpflug system (Pentacam; Oculus Optikgeräte GmbH, Wetzlar, Germany) and IOP measurements.

To evaluate intra-operator variability (repeatability) two experienced ophthalmologists per site performed all IOP assessments. The examiner was masked to the measurements taken and a different observer read and recorded the IOP readings. IOP was measured once with GAT and twice by ART (ART1, ART2) with a resting period of 5 minutes between each set of measurements. The order between GAT, ART1 and ART2 measurements was randomized using one of the following sequences:

A. ART1, GAT, ART2;
B. GAT, ART1, ART2;
C. ART1, ART2, GAT.

A randomisation list of the sequences (by means of a list of random numbers) common to both centres was generated. When an eligible subject was enrolled in the study, a randomisation code was assigned from the list.

Reproducibility was evaluated on a second session: three evaluators per site were designated (A, B, C) and two of them were chosen at random (A and B, B and C, A and C) to perform two measurements each in random order (i.e. ABAB or BABA) on the same patient with a 5-minute break between testing. These measures were numbered from 3 to 6 in order to clarify that they were taken outside the part of the study on intra-operator variability. Therefore measures 3 and 5 were taken by the same operator as well as 4 and 6.

### IOP measurements

Both centres were given specific details regarding the study protocol and were instructed to respect the standard operating procedures for IOP measurements approved by the European Vision Institute. During the measurements, the operators made every effort to reduce or eliminate any possible source of avoidable variability. Since 4 IOP records among POAG patients exceeded ±3 standard deviations (SD), they were defined as outliers and excluded from analyses.

#### Goldmann applanation tonometry

At each centre, the same tonometer was used throughout the study, and the calibration was checked every measurement day. GAT was performed using a method which has been largely validated in previous studies by our group. After instillation of benoxinate hydrochloride 0.4% drops and fluorescein sodium 1 mg strips, two measurements were taken; if the two measurements differed by 2 mmHg or more a third measurement was taken. The mean of two or the median of 3 recordings was used for analysis. The operator who measured IOP by GAT was experienced and instructed not to read the value, which was collected by a second operator [17,18].

#### Servo-controlled Bioresonator applanation resonance tonometry®

Servo-controlled Bioresonator ART® is based on resonance technique: as the ART sensor automatically and objectively applanated the cornea, the acoustic impedance of the cornea mechanically loaded the sensor forming an oscillating system with a new resonance frequency; a force transducer enabled the calculation of the IOP according to Imbert-Fick’s law. It has been suggested that a convex sensor tip and an aiming light may provide easier applanation and more accurate measurements than those without [3,13,15]. One drop of benoxinate hydrochloride 0.4% was instilled before ART. Since an analysis of the SD for different numbers of measurements revealed a similar SD for three measurements compared to six, three measurements with ART were considered sufficient [14]: each measurement with ART was the mean of three consecutive applanations against cornea. The sampled data were automatically processed and the median value of the repeated measurements was displayed with a Q-value (qualitative score of measurement: Q = 1, optimum; Q = 2, acceptable; Q = 3 or 4, questionable; Q = 5, low quality) A qualitative score of Q1 or Q2 was recommended; if Q ≥ 3, the measurement was repeated and reported on the case report form (CRF).

### Statistical analysis

Statistical analysis was performed with SPSS software (version 20.0, SPSS Inc., Chicago, IL).

IOP measurements were expressed as mean ± SD. Paired *t*-test was carried out to assess whether there were any differences between IOP measurements taken using GAT and Bioresonator ART®. A value of *p* < 0.05 was considered statistically significant. The agreement between GAT and ART readings and between ART1 and ART2 readings was assessed with the Bland-Altman method, in which the differences between readings were plotted with the mean measurements. Repeatability and reproducibility were assessed by coefficient of variation (CoV) and intraclass correlation coefficient (ICC), based on 2-way random-effects analysis of variance. ICC is defined as the ratio of the between-subjects variance to the sum of the pooled within-subject variance and the between-subjects variance; ICC agreement is commonly classified as follows: perfect if ICC = 1, almost perfect 0.81 to 0.99, substantial 0.61 to 0.80, moderate 0.41 to 0.60, fair 0.21 to 0.40, slight 0.01 to 0.20 and poor −1 to 0 [19,20]. CoV is calculated as the pooled within-subject SD divided by the mean of the measurements and expressed as a percentage. Multiple regression analysis was used to assess the influence of ocular structural factors (central corneal thickness, keratometry, axial length), age, diagnosis, operator and randomization sequence on the measurement differences.

## Results

Table 1 summarises the demographic and ophthalmic characteristics of the study participants. In the POAG and control groups respectively, mean age was 69.1 ± 9.4 and 68.9 ± 9.6 years; F/M ratio 58/57 and 36/27; central corneal thickness (CCT) 540 ± 33 and 547 ± 27 μm; keratometry 44.27 ± 1.61 and 43.90 ± 2.07 D, axial length 23.43 ± 1.25 and 23.59 ± 1.68 mm (p > 0.18).

**Figure 1:**
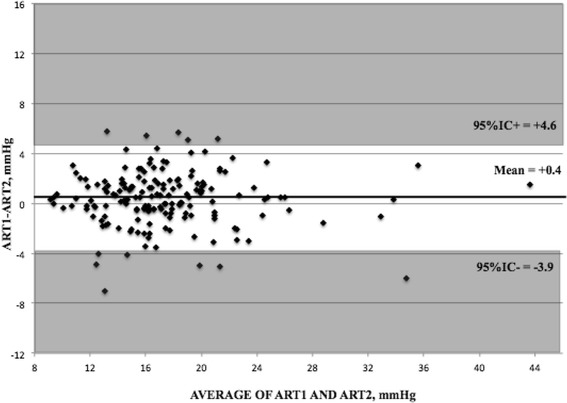
**Bland-Altman plot for the first measurement using applanation resonance tonometry (ART1) and the second measurement using ART (ART2).**

Table 2 shows mean IOP data. IOP readings with both GAT and ART were significantly higher in treated POAG patients than in controls (p ≤ 0.05). In the entire cohort, ART1 was 0.4 ± 2.2 mmHg (−7.0 to 5.7 mmHg) higher than ART2 (p = 0.03); no significant differences were found within groups (p = 0.64). Differences between means and medians were small and did not change significantly when outliers were considered or not. Test-retest difference with ART fell within ±1 mmHg in 41% of cases, within ±2 mmHg in 70%, within ±3 mmHg in 85%. 15% had a test-retest difference higher than ± 3 mmHg. Bland-Altman plot is shown in Figure 1; 95% intervals of confidence were −3.9 and +4.6 mmHg.

**Table 2:** **Mean data on intraocular pressure**

	**Overall**	**Normal**	**Glaucoma**	***p***
**ART1**	17.6 ± 5.0 (9.3, 44.4)	16.6 ± 3.2 (9.5, 26.2)	18.1 ± 5.7 (9.3, 44.4)	0.027*
**ART2**	17.2 ± 5.1 (9.0, 42.9)	16.1 ± 3.4 (9.3, 25.8)	17.8 ± 5.7 (9.0, 42.9)	0.016*
**GAT**	15.9 ± 4.3 (8.5, 39.5)	15.2 ± 2.6 (9.5, 22.5)	16.3 ± 5.0 (8.5, 39.5)	0.05*
**ART1 - ART2**	0.4 ± 2.2 (−7.0, 5.7)	0.5 ± 2.2 (−7.0, 5.7)	0.3 ± 2.1 (−6.0, 5.6)	0.636
**ART1 - GAT**	1.7 ± 3.1 (−5.8, 11.4)	1.4 ± 2.9 (−4.5, 9.2)	1.8 ± 3.1 (−5.8, 11.4)	0.347
**ART2 - GAT**	1.3 ± 3.1 (−5.7, 10.6)	0.9 ± 3.0 (−5.7, 8.8)	1.5 ± 3.2 (−5.5, 10.6)	0.211

All data were mean ± SD (range), mmHg.

*p ≤ 0.05 (normal versus glaucoma).

ART, applanation resonance tonometry; GAT, Goldmann applanation tonometry.

**Figure 2:**
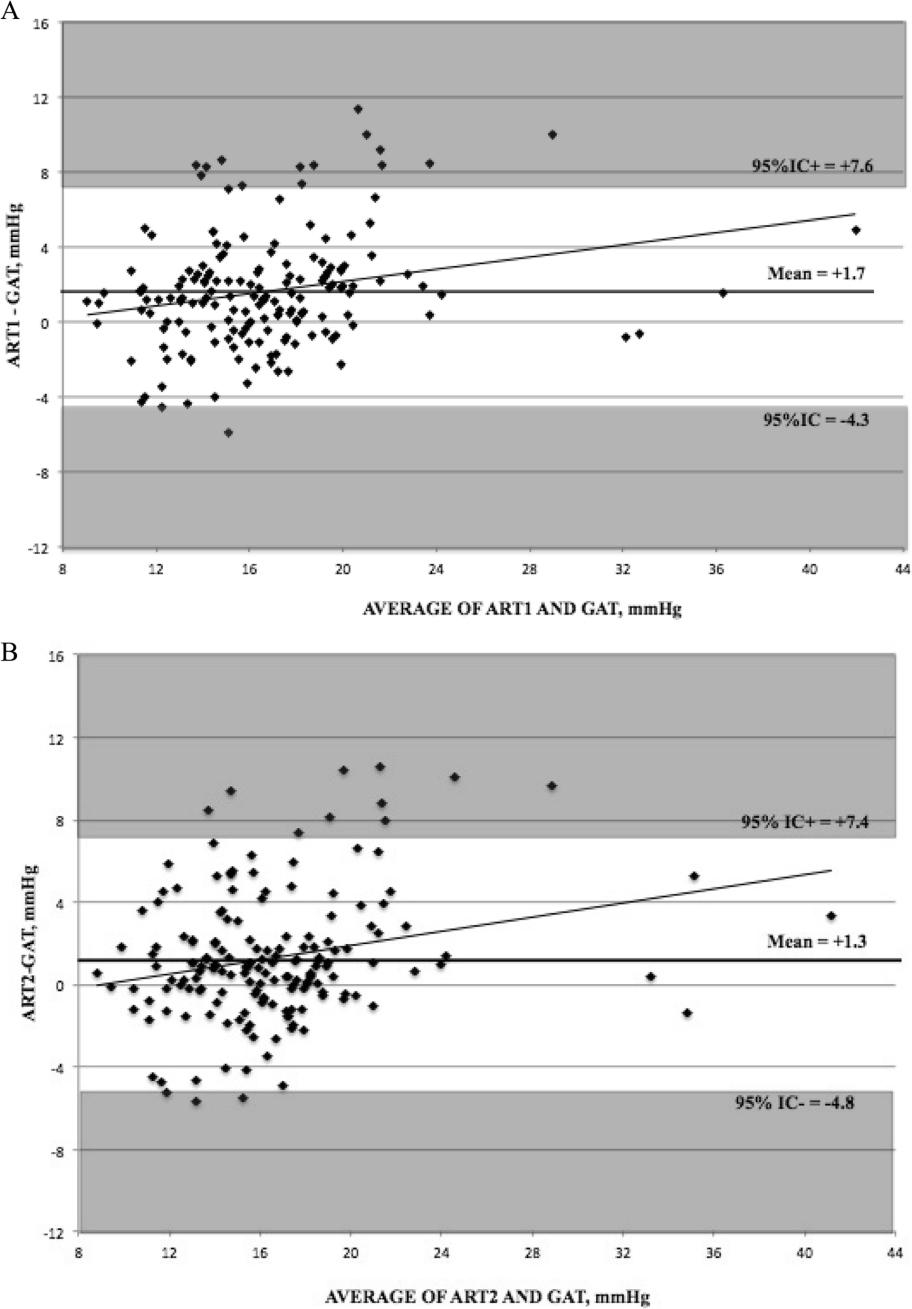
**Bland-Altman plots for (A) the first measurement using applanation resonance tonometry (ART1) and Goldmann applanation tonometry (GAT) and (B) the second measurement using ART (ART2) and GAT.**

ART1 and ART2 were respectively 1.7 ± 3.1 (−5.8 to 11.4) and 1.3 ± 3.1 (−5.7 to 10.6) mmHg higher than GAT (p ≤ 0.01). The difference between ART and GAT significantly increased with increasing mean IOP (Figure 2A and B), whereas sequence had no significant effects on results (Table 3). We also calculated the regression formula for the difference between the mean (x) and the difference (y) of GAT and, respectively, ART1 and ART2, which were: y = 0.1631x – 1.07, p = 0.01, R^2^ = 0.06 and y = 0.172x - 1.543, p < 0.001, R^2^ = 0.06.

**Table 3:** **Effect of sequence on the whole population study**

	**Sequence A**	**Sequence B**	**Sequence C**
	**(ART1, GAT, ART2)**	**(GAT, ART1, ART2)**	**(ART1, ART2, GAT)**
**ART1**	16.7 ± 4.4	17.9 ± 4.6	18.1 ± 5.9
**ART2**	16.0 ± 5.0	17.7 ± 4.2	18.0 ± 5.7
**GAT**	15.3 ± 4.0	15.9 ± 3.2	16.5 ± 5.4
**ART1 - ART2**	0.6 ± 1.9	0.3 ± 2.0	0.2 ± 2.5
**ART1 - GAT**	1.4 ± 3.0	2.0 ± 3.3	1.6 ± 2.9
**ART2 - GAT**	0.8 ± 3.2	1.8 ± 3.3	1.4 ± 2.9

All data were mean ± SD, mmHg. No significant differences were found between sequences (p >0.05).

ART, applanation resonance tonometry; GAT, Goldmann applanation tonometry.

We inspected possible sources of variability with multivariate analysis, and only ART value was found to significantly affect the difference between GAT and ART values (Table 4).

**Table 4:** **Multivariate regression analysis between the demographic and ophthalmic characteristics of the study participants and the difference between ART and GAT readings (** ***p*** **values)**

	**ART1 - GAT**	**ART2 – GAT**
Age	0.77	0.88
Diagnosis	0.21	0.22
Central corneal thickness	0.37	0.12
Keratometry	0.24	0.16
Axial lenght	0.24	0.16
ART1	0.05*	0.27
ART2	0.15	0.00*
GAT	0.14	0.06
Sequence	0.28	0.36

*p ≤ 0.05.

**Figure 3:**
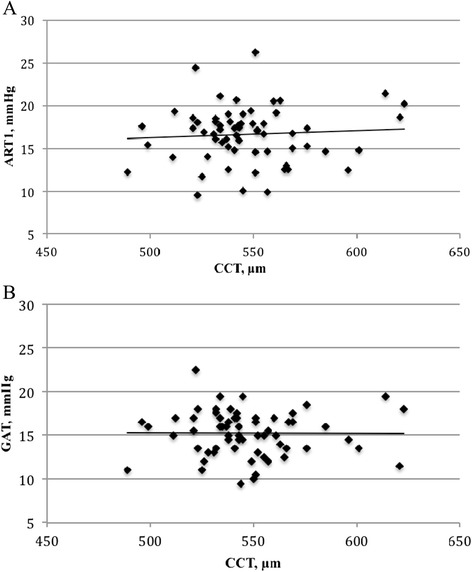
**Scatter plots for (A) the first measurement using applanation resonance tonometry (ART1) and (B) Goldmann applanation tonometry (GAT) versus central corneal thickness (CCT) in the normal group.**

Regression analysis between CCT and either GAT or ART were negligible in the whole population (p = 0.52 and 0.55; R^2^ = 0.004 and 0.002, respectively). On the subgroup of normal subjects, we found no association and negligible correlation between CCT and IOP with both GAT (p < 0.001; R^2^ = 0.00) and ART (p < 0.001, R^2^ = 0.00) (Figure 3).

Data on intra-operator variability are given on Table 5. In the entire cohort, ART intra-operator CoV was 7.0 ± 6.3 % with no significant differences between POAG and normal subjects (respectively 6.6 ± 5.7 % and 7.6 ± 7.3 %; p = 0.37); little variation was found when sub-analyses were performed for sequence and diagnosis (range: 5.7-7.9%). Intra-operator ICC was almost perfect in the entire cohort (0.90-0.91) and in glaucoma patients (0.91-0.93), and substantial in normal subjects (0.77-0.81).

**Table 5:** **ART repeatability: intra-operator coefficient of variation (mean ± SD; 95% IC) and intraclass correlation coefficient**

**Coefficient of variation (%)**				**Intraclass coefficient of correlation**		
	**Overall**	**Normal**	**Glaucoma**	**Overall**	**Normal**	**Glaucoma**
**Overall**	7.0 ± 6.3 (−5.3;19.3)	7.6 ± 7.3 (−6.7;21.6)	6.6 ± 5.7 (−4.6;17.8)	0.91	0.80	0.92
**Sequence A**	6.7 ± 5.6 (−4.3;17.7)	7.8 ± 5.8 (−3.6;19.2)	6.2 ± 5.5 (−4.6;16.3)	0.91	0.77	0.92
**Sequence B**	6.3 ± 6.7 (−6.8;19.4)	7.1 ± 8.5 (−9.6;23.8)	5.7 ± 5.1 (−4.3;15.1)	0.90	0.81	0.93
**Sequence C**	7.9 ± 6.6 (−5.0;20.8)	7.8 ± 7.4 (−6.7;22.3)	7.9 ± 6.2 (−4.3;20.1)	0.90	0.79	0.91

**Figure 4:**
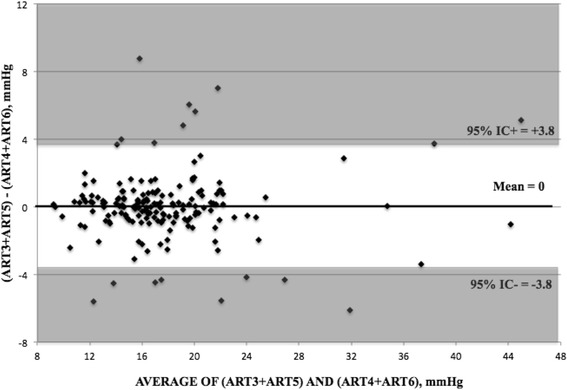
**Bland-Altman plots for the measurements taken by one operator (ART3 + ART5) and the measurements taken by the other one (ART4 + ART6).**

Data on inter-operator variability are given on Table 6. Overall CoV was 6.0 ± 10.9% (comparing ART3 vs ART4) and 6.1 ± 6.3% (ART5 vs ART6); this corresponded to ICC of 0.95 and 0.93, respectively. No significant differences were shown for diagnosis (POAG vs normal), operators and sequence (ABAB vs BABA). The inter-operator test-retest variability for ART was negligible (p = 0.95). Bland-Altman plot is given in Figure 4.

**Table 6:** **ART reproducibility: inter-operator coefficient of variation (mean ± SD; 95% IC) and intraclass correlation coefficient in the whole study population**

	**Coefficient of variation (%)**			**Intraclass coefficient of correlation**		
	**Overall**	**Normal**	**Glaucoma**	**Overall**	**Normal**	**Glaucoma**
**ART3 vs ART4**	6.0 ± 10.9 (−15.4;27.4)	5.8 ± 7.1 (−8.1;19.7)	6.1 ± 12.6 (−18.6;30.8)	0.95	0.86	0.97
**ART5 vs ART6**	6.1 ± 6.3 (−6.2;18.4)	5.7 ± 6.1 (−6.3;17.7)	6.4 ± 6.5 (−6.3;19.1)	0.93	0.88	0.94
**ART3 + ART5 vs ART4 + ART6**	8.0 ± 6.8 (−5.3;21.3)	7.6 ± 6.0 (−4.2;17.8)	8.2 ± 7.2 (−5.9;22.3)	0.94	0.87	0.94

“ART3 + ART5 versus ART4 + ART6” compared all measurements taken by one investigator versus those taken by the other one.

## Discussion

Our study focused on the reproducibility of servo-controlled Bioresonator ART®, which is a poorely explored topic. We used a study design which has been previously validated and used to test another new tonometer, Pascal, versus GAT [21]. In this paper, we showed that ART had high repeatability (ICC ≥ 0.80, CoV of 6.6-7.6 %) and high reproducibility (ICC ≥ 0.86, CoV of 5.7-8.2%) in both glaucoma patients and controls.

Intra-operator variability was almost perfect in the entire cohort and in glaucoma subgroup, and substantial in normal subjects. ART1 was 0.4 ± 2.2 mmHg (−7.0 to 5.7 mmHg) higher than ART2 (p = 0.03). It is likely that a tonometric effect, which has been shown for all contact tonometries (including non-applanating and non-indentating devices) [22] may contribute in explaining this finding. It is also possible that normal subjects, being not familiar with IOP measurements, were apprehensive at first IOP record and more relaxed at the second one [3], thus providing lower readings.

Repeatability values were similar to those reported by Salvetat et al. who assessed an excellent ART intra-operator variability (ICC ≥ 0.95, CoV range 2-17%), although significantly lower than GAT’s [16]. The comparison of reproducibility between GAT and ART was outside the scope of this paper. In the literature, a high reproducibility is reported for GAT: inter-observer variation coefficient = 4.6% [23]; CoV = 9% and ICC = 0.82 [24]; inter-observer mean difference = −0.8 ± 3.9 mmHg [25]. In theory, a fully-automated system as ART should have a better reproducibility than GAT, in which the operator manually regulates the applanation force and interprets an optical pattern.

Our analysis showed that ART exceeded GAT’s measurements by a mean of 1.3-1.7 mmHg, thus confirming previous findings [16]. Differences between GAT and other tonometers may be explained by the different size of the applanating probes, and by different tonometric strategies (i.e. applanation, indentation, rebound, dynamic contour).

In literature GAT repeatability was high in glaucoma patients (intra-observer correlation coefficient of 0.989) and good in controls (CoV = 9.7% and ICC = 0.79) [23,24]. Previous studies on GAT intra-operator repeatability reported 95% confidence interval of 2.5 mm Hg [26]; as a general rule, in presence of GAT measures exceeding 2 mm Hg, other factors than variability should be considered. On our dataset, we found that 41% of ART test-retest measures fell within 1 mmHg and 70% within 2 mmHg, thus 30% were 2 mmHg or more.

In this study, we excluded 4 outliers from the group of glaucoma, who had readings exceeding 3 SD. These cases had Q-values of 1 or 2 (as defined in Methods) and no procedural deviations or abnormal eye characteristics were found. One case was excluded as GAT measurement had not been reported on data sheet. Demographic and ophthalmic characteristics of excluded patients are shown in Table 7. Even if treated glaucoma patients did not have normal distributions (the distribution, as expected, had a flatter shape without kurtosis), their data were suggestive of sporadic measurement errors. In any case, it should be noted that the difference between the two tonometries ranged from −5.8 to 11.4 mmHg, thus suggesting that they may not be interchangeable in a clinical setting.

**Table 7:** **Demographic and ophthalmic characteristics of excluded patients**

	**Age, years**	**Sex (F/M)**	**Keratometry, diopters**	**Axial lenght, mm**	**CCT, μm**	**ART1**	**Q**	**ART2**	**Q**	**GAT**	**Sequence**	**POAG/control**
*75	71	F	44.85	23.21	531	33.5	1	21.23	1	31	A	POAG
*77	61	M	42.83	23.92	548	51.1	2	51.1	1	38	A	POAG
*104	57	M	43.47	24.3	533	25.5	2	43.1	2	25.5	A	POAG
*146	68	F	43.66	24.12	508	18.4	1	9	1	12	C	POAG
*155	54	F	43.31	24.6	543	20.8	1	14.8	2	MISSING	C	POAG

*patient number.

We inspected sources of difference between ART and GAT and found out that differences tended to increase at higher IOP values (Figure 2A and B) as previously described [16]. Yet, R^2^ values were considerably low, a fact that may indicate that regression model may be a poor descriptor of study data, in particular only a minority of measurements was higher than normal. A properly designed study to explore the characteristics of ART in eyes with IOP higher than 21 mmHg is recommendable, also considering that in a previous paper ART failed to respect the ISO standards for patients with IOP > 23 mmHg, when compared to GAT [14].

ART previously received validation by two manometric studies on *in vitro* porcine-eye models: precision (expressed as standard deviation) was acceptable according to both Eklund et al. (±0.94 mmHg) [2] and Hallberg et al. (±1.03 mmHg) [15]. Additional studies, especially of human intracameral manometry, are needed to further assess how corneal properties can influence ART IOP measures.

In the group of normal subjects, we found no significant association (and poor correlation) between CCT and IOP with both GAT and ART, which is, at least for GAT, an unexpected result. Our data are in contrast with two studies showing an influence of CCT on ART [2,16]; in particular, we cannot confirm the findings by Salvetat et al. who suggested a similar behaviour of both tonometers errors to the CCT (being the relationship between ART-GAT values and CCT not significant) [16]. A possible explanation of our results could be the fact that our sample is not representative of the normal population due to the small size. It should be pointed out that both CCT and ART measures in this study were fully automated and operator-independent. Yet, we measured CCT using Pentacam, which is not the standard (even if there are reports suggesting that it correlates well with the gold standard, ie ultrasound pachimetry [27,28]). Further studies are needed to better understand the relationship between CCT and IOP measurements, in particular those obtained with ART, as it is complex and incompletely determined in both normal subjects and glaucoma patients [29-31].

GAT is the standard in clinical practice and in the management of glaucoma. Still, it is largely influenced by ocular properties and variations in corneal biomechanics [18]; it is subjective and prone to learning; its use outside clinical settings is limited by non-portability and by the need of topical anesthetic, fluorescein and slit-lamp microscope to perform measurement. Great efforts have been made to develop other accurate and objective tonometers; among them, ICare rebound tonometer (RBT, Tiolat, Helsinki, Finland) and Pascal dynamic contour tonometer (DCT, Swiss Microtechnology AG, Port, Switzerland) showed high reproducibility and less dependency to ocular characteristics [21,23,25].

## Conclusions

In this paper, we confirmed that reproducibility and repeatability is high also for ART: inter-examiner reproducibility was almost perfect when subjects were assessed as a whole or in subgroups and intra-examiner repeatability was excellent, although decreasing at high IOP levels. ART significantly overestimated GAT measurements, especially at higher IOP ranges; GAT and ART were significantly influenced only by mean ART value and not by corneal properties. Together with other characteristics of ART (it is objective; operator-independent; as time-consuming as GAT; it does not require fluorescein strips; measurement is the mean of 3 readings in improve quality; a quality score is also given), the good performance on reproducibility may suggest a possible use in clinical practice, even if larger clinical evaluations and validation with manometric data are mandatory.

## Abbreviations

ART: Applanation resonance tonometry
BCVA: Best-corrected visual acuity
CCT: Central corneal thickness
CoV: Coefficient of variation
CRF: Case report form
GAT: Goldmann applanation tonometry
ICC: Intraclass correlation coefficient
IOP: Intraocular pressure
POAG: Primary open-angle glaucoma.
